# Deterioration of bladder compliance after botulinum toxin A injection and discontinuation of medication for overactive bladder

**DOI:** 10.1002/iju5.12496

**Published:** 2022-06-20

**Authors:** Naoki Wada, Mayumi Ishikawa, Masaya Nagabuchi, Shogo Makino, Kotona Miyauchi, Noriyuki Abe, Hidehiro Kakizaki

**Affiliations:** ^1^ Department of Renal and Urologic Surgery Asahikawa Medical University Asahikawa Japan

**Keywords:** botulinum toxin type A, low compliance bladder, overactive bladder

## Abstract

**Introduction:**

We report a case of deterioration of bladder compliance after botulinum toxin type A injection and discontinuation of medication for overactive bladder.

**Case presentation:**

A female patient with overactive bladder in her sixties had been visiting our outpatient clinic regularly for 4 years. She had received posterolateral spondylus fusion twice, which resulted in a compression fracture. She had been receiving a combination therapy of anticholinergics and β3‐adrenoceptor agonist for the management of overactive bladder. She received botulinum toxin type A injection for refractory overactive bladder and discontinued medical treatment for overactive bladder. Three months after botulinum toxin type A injection, cystometry revealed the deterioration of bladder compliance. Renal dysfunction, hydronephrosis, and vesicoureteral reflux were shown. Renal function and hydronephrosis were improved after restarting anticholinergics and β3‐adrenoceptor agonist therapy and inserting a temporary transurethral catheter.

**Conclusion:**

Deterioration of bladder compliance may occur after botulinum toxin type A injection and discontinuation of overactive bladder medication in some patients with underlying neurological disease.

**Abbreviations & Acronyms**
BTX‐A: botulinum toxin type A
CMG: cystometrogram
CT: computed tomography
DO: detrusor overactivity
FDV: first desire to void
NDV: normal desire to void
OAB: overactive bladder
Pabd: abdominal pressure
Pdet: detrusor pressure
Pves: vesical pressure
SDV: strong desire to void
VCUG: voiding cystourethrography
VUR: vesicoureteral reflux

**Keynote message**

Discontinuation of preceding medications for overactive bladder, including anticholinergics and β3‐adrenoceptor agonist, after botulinum toxin type A (BTX‐A) treatment may reveal the pre‐existing low compliance bladder in patients with underlying neurological disease. It is necessary to be attentive to the possibility that some patients need to continue anticholinergics and/or β3‐adrenoceptor agonist even after BTX‐A treatment to prevent deterioration of bladder compliance.

## Introduction

Refractory OAB is defined as the condition where patients have tried one or more anticholinergics and/or β3‐adrenoceptor agonist drugs for urgency and urgency leakage, yet their condition remains unimproved due to insufficient efficacy or intolerable side‐effects. Intradetrusor injection of BTX‐A inhibits acetylcholine release and suppresses involuntary contraction of the bladder.^1^ The efficacy of BTX‐A injection is well established.^2^ Herein, we present a case of deterioration of bladder compliance after BTX‐A injection and discontinuation of medication for OAB.

### Case report

**Fig. 1:**
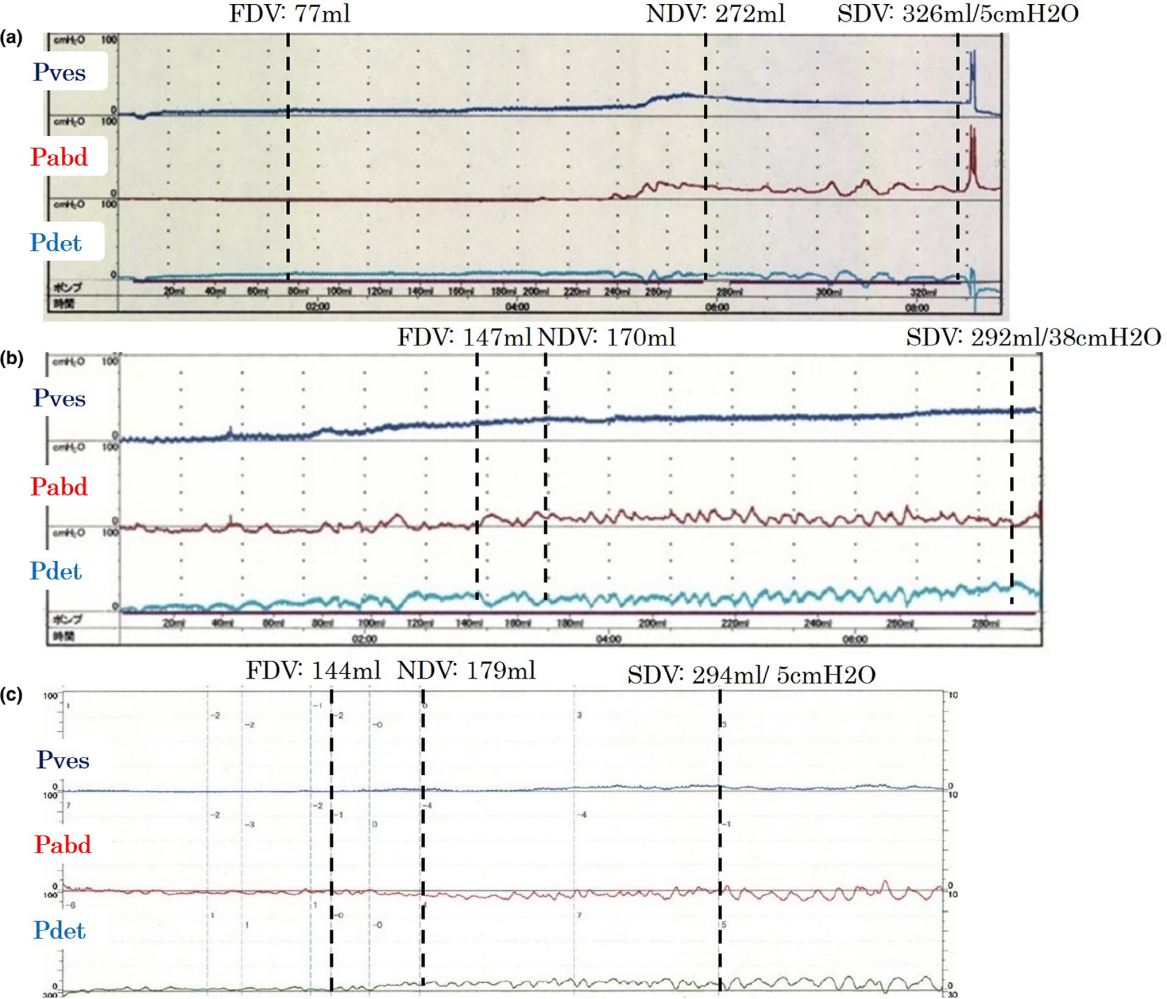
Cystometrogram before and after BTX‐A injection. (a) Cystometry before BTX‐A injection under administration of anticholinergics and β3‐adrenoceptor agonist therapy showed no DO and 5 cmH_2_O of Pdet at 326 mL of maximum cystometric bladder capacity (compliance = 65 mL/cmH_2_O). (b) Cystometry performed again at 3 months after BTX‐A injection showed no DO and 38 cmH_2_O of Pdet at 292 mL of bladder capacity (compliance = 7.7 mL/cmH_2_O). (c) Cystometry performed after restarting β3‐adrenoceptor agonist and anticholinergics therapy and inserting a temporary transurethral catheter showed no DO and 5 cmH_2_O of Pdet at 294 mL of bladder capacity (compliance = 59 mL/cmH_2_O).

**Fig. 2:**
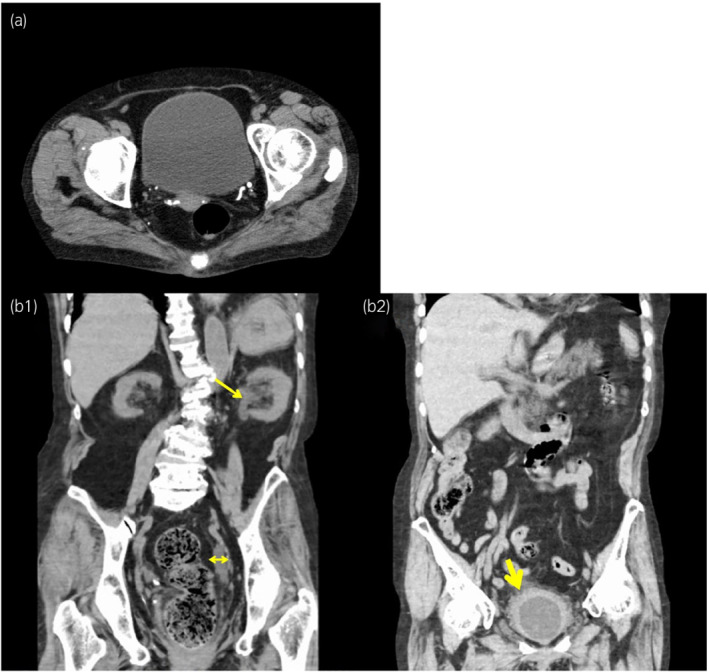
Serial CT images. (a) A CT image obtained at 9 months before BTX‐A injection showed no bladder wall thickness. (b) CT image obtained at 3 months after BTX‐A injection showed left hydronephrosis (arrow), ureteral dilatation (double‐headed arrow) (b1), and bladder wall thickness (big arrow) (b2).

This case report focuses on a female patient in her sixties suffering from OAB symptoms with histories of chronic rheumatoid arthritis and Sjogren syndrome. The patient had been visiting our outpatient clinic regularly for 4 years. She received lumbar posterolateral spondylus fusion twice for severe lumber spinal canal stenosis 9 and 13 years ago and experienced a compression fracture 2 years ago. She had been receiving a combination therapy of anticholinergics (fesoterodine) and β3‐adrenoceptor agonist (mirabegron) for OAB symptoms, but anticholinergics therapy was sometimes discontinued due to dry mouth. This approach did not significantly improve OAB symptoms. The mean and maximum voided volume on frequency‐volume charts were below 100 and 140 mL, respectively, with 20–40 mL of postvoid residual urine. Cystometry before BTX‐A injection under administration of anticholinergics and β3‐adrenoceptor agonist showed no DO and 5 cmH_2_O of Pdet at 326 mL of maximum cystometric bladder capacity (compliance = 65 mL/cmH_2_O) (Fig. 1a). The bladder wall was not thick on CT scan (Fig. 2a). We administered 100 units of BTX‐A into the bladder wall to manage refractory idiopathic OAB. At the same time, anticholinergics and β3‐adrenoceptor agonist were discontinued. Bladder mucosal abnormalities, such as Hunner lesion, were not detected on cystoscopy.

**Fig. 3:**
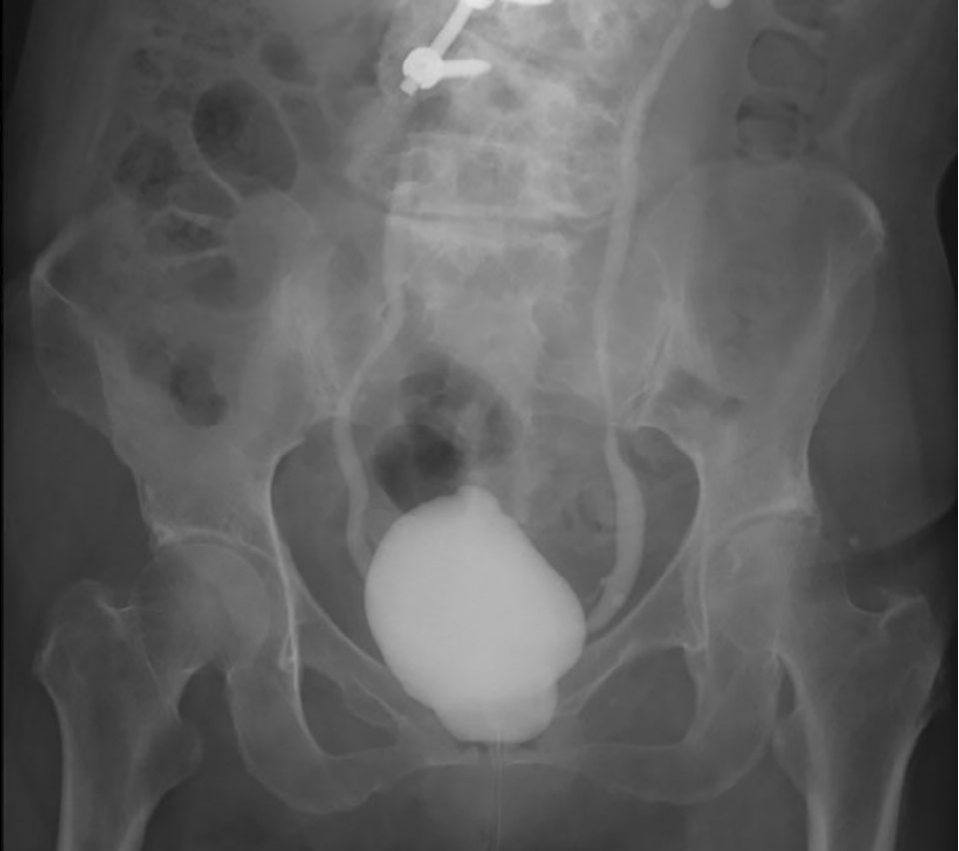
VCUG performed at 3 months after BTX‐A injection showed bilateral moderate VUR.

**Fig. 4:**
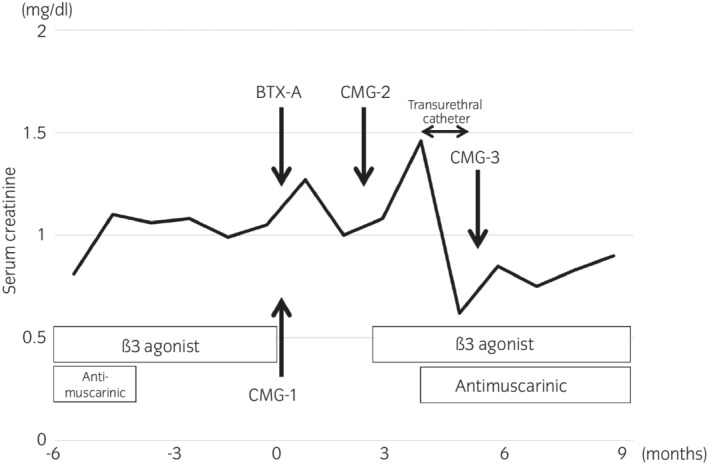
Scheme of the clinical course of the patient. The patient had received a combination treatment of β3‐adrenoceptor agonist and anticholinergics for the management of OAB symptoms. Only β3‐adrenoceptor agonist had been used without anticholinergics due to dry mouth for 3–4 months before BTX‐A injection. At the time of BTX‐A injection, cystometry showed normal bladder compliance (Fig. 1a) (CMG‐1). Three months after BTX‐A injection, cystometry demonstrated the deterioration of bladder compliance (Fig. 1b) (CMG‐2) and only β3‐adrenoceptor agonist was resumed. Following 1 month, renal dysfunction and bilateral VUR were noted. Anticholinergic therapy was also resumed, and a temporal urethral catheter was inserted for 6 weeks, which improved renal function and bladder compliance (Fig. 1c) (CMG‐3).

Three months after BTX‐A injection, her OAB symptoms were poorly improved with only a slight improvement in urinary frequency. OAB symptom score was changed from 13 (Q1: 2, Q2: 3, Q3: 4, Q4: 4) to 9 (Q1: 1, Q2: 3, Q3: 3, Q4: 2). Cystometry showed no DO and 38 cmH_2_O of Pdet at 292 mL of maximum cystometric bladder capacity (compliance = 7.7 mL/cmH_2_O) (Fig. 1b). Mirabegron was resumed, but a few weeks later, serum creatinine level was elevated from 1.05 to 1.46 mg/dL. At this time, a CT scan showed hydronephrosis and bladder wall thickness. Continuous pyuria was found in this patient both before and after BTX‐A injection. Bilateral VUR was noted on VCUG (Fig. 3). Serum creatinine level and hydronephrosis were immediately improved after restarting anticholinergics therapy and inserting a temporary transurethral catheter for a period of 6 weeks (Fig. 4). Bladder compliance was also normalized at that point (Fig. 1c). After this episode, the patient received BTX‐A injection twice (100 and 200 units, respectively) again with concurrent anticholinergics and β3‐adrenoceptor agonist therapies, and no hydronephrosis or renal dysfunction was developed.

## Discussion

Herein, we present a case of deterioration of bladder compliance and upper urinary tract after BTX‐A injection and discontinuation of medication for OAB. It is speculated that a combination therapy of anticholinergics and β3‐adrenoceptor agonist plays a significant role in maintaining bladder compliance even after BTX‐A treatment in this patient. The key messages in this case report are that deterioration of bladder compliance and upper urinary tract may occur after BTX‐A injection and discontinuation of OAB medication in some patients with underlying neurological diseases.

Assessment of the bladder wall thickness after BTX‐A injection might indicate the possibility of bacterial infection of the bladder. However, continuous pyuria was present in this patient both before and after BTX‐A injection. Thus, we speculated that this change was independent of bacterial infection.

The Japanese phase III, randomized, double‐blind, placebo‐controlled trial in OAB patients who had been inadequately managed with medications demonstrated the BTX‐A group had significantly greater decreases in the mean number of daily urinary incontinence episodes, number of episodes of micturition, urgency, and nocturia from the baseline compared with the placebo group without preceding OAB medication.^2^ In real‐world practice, most patients receiving BTX‐A injections discontinue preceding OAB medication after BTX‐A treatment. The large real‐world data on BTX‐A efficacy and safety of treatment showed that only 52 of 504 patients (10.3%) intended to continue using OAB medications following BTX‐A treatment, and very few patients actually continued OAB medication.^3^ Discontinuation of preceding medication for OAB, including anticholinergics and β3‐adrenoceptor agonist, after BTX‐A treatment may reveal the pre‐existing low compliance bladder in some patients with underlying neurological disease.

BTX‐A injections improve bladder compliance in some patients with neurogenic low compliance bladders who are refractory to oral medications.^4^, ^5^, ^6^ However, it seems that the effectiveness of this method is lower in both adults and children with spinal disorders and low compliance bladder.^4^, ^5^, ^6^ O'Connor *et al*. evaluated patients with refractory impaired bladder compliance secondary to spinal myelopathy treated with 300 units of BTX‐A injection.^4^ Bladder compliance was improved only in 22 of 71 (31%) patients, leaving the remaining patients (69%) as non‐responders. Peyronnet *et al*. also demonstrated that all urodynamic parameters, including bladder compliance, were significantly improved after BTX‐A injection in patients with spina bifida; however, the clinical success rate was lower in patients with low compliance bladder.^5^ Danacioglu *et al*. reported that children with a neurogenic bladder due to myelomeningocele, who had low compliance bladder without DO, showed poor therapeutic responses.^6^ Thus, the effectiveness of BTX‐A therapy for low compliance bladder is limited, and it may depend on the baseline bladder compliance.

It is unclear which of the β3‐adrenoceptor agonist, anticholinergics, or their combination contributed the most to improving bladder compliance and renal function after the insertion of a temporal transurethral catheter. We speculated that the β3‐adrenoceptor agonist contributed more to the improvement in bladder compliance, because deterioration of bladder compliance and renal function had not been detected when using only the β3‐adrenoceptor agonist (without anticholinergics due to dry mouth symptoms) for several months before BTX‐A injection (Fig. 4). Several studies have reported supportive findings regarding the efficacy of monotherapy or the combination therapy of β3‐adrenoceptor agonist with anticholinergics for neurogenic low compliance bladder.^7^, ^8^, ^9^

We did not perform cystometry or VCUG when we first evaluated this patient. In addition, it may be necessary to perform urodynamic evaluation in OAB patients with underlying neurological disease. Bladder compliance and upper urinary tract should be monitored in patients with neurogenic OAB who receive BTX‐A injections and discontinue preceding OAB medications.

## Conclusion

Patients with underlying neurological diseases may need to continue anticholinergics and/or β3‐adrenoceptor agonist therapy even after BTX‐A injections to prevent deterioration of bladder compliance and upper urinary tract.

## Author contributions

Naoki Wada: Conceptualization; data curation; writing – original draft. Mayumi Ishikawa: Investigation; resources. Masaya Nagabuchi: Investigation; resources. Shogo Makino: Investigation; resources. Kotona Miyauchi: Investigation; resources. Noriyuki Abe: Investigation; resources. Hidehiro Kakizaki: Supervision; writing – review and editing.

## Conflict of interest

The authors declare no conflict of interest.

## Approval of the research protocol by an Institutional Reviewer Board

Not applicable.

## Informed consent

Not applicable.

## Registry and the Registration No. of the study/trial

Not applicable.
